# Hong Kong Children’s School Readiness in Times of COVID-19: The Contributions of Parent Perceived Social Support, Parent Competency, and Time Spent With Children

**DOI:** 10.3389/fpsyg.2021.779449

**Published:** 2021-12-01

**Authors:** Eva Yi Hung Lau, Jian-Bin Li

**Affiliations:** Department of Early Childhood Education, The Education University of Hong Kong, Hong Kong, Hong Kong SAR, China

**Keywords:** school transition, school readiness, parents, family, COVID-19, pandemic

## Abstract

School readiness is an important but challenging issue of child development, especially during COVID-19 when most of the traditional offline activities that could promote school readiness (e.g., on-site visit) have been canceled. There is a gap between the knowledge needed to promote children’s school readiness in times of pandemic and the limited understanding of this topic so far. This gap could be particularly concerning in the social contexts where examinations are stressed and educational competition is high (e.g., Hong Kong). In this study, we examined how well children were ready for primary school, the extent to which parent perceived social support was related to children’s school readiness, and whether parent competence and their time spent with children would moderate the said link. A cross-sectional design survey with total population sampling (supplemented with convenience sampling) was conducted. Massive e-mails were sent to all kindergartens in Hong Kong inviting them to join the study by distributing the survey link to the parents of their K3 students. A total of 643 Hong Kong parents whose children were about to transition to primary school (87.1% mother) participated, answering measures specifically designed for this study online about how well they thought their children were ready for school, their competence to help with children’s school transition, and how much time they spent with children. Data were analyzed with PROCESS macro (model 3) in SPSS. The results found that most parents considered that their children were not fully ready for school, especially in terms of academic skills, self-management, and mental preparation. Furthermore, moderation analyses showed that after controlling for a number of demographic variables, parent perceived social support was positively related to better school readiness in children and this link was jointly moderated by parent competence and time spent with children. Specifically, children were rated most ready when parent perceived stronger social support, felt more competent, and spent more time with children. By contrast, the link between perceived social support and children’s school readiness was insignificant for parent who felt more competent but spent less time with children. Implications of how to enhance children’s school readiness are discussed.

## Introduction

The COVID-19 pandemic has considerably disrupted children’s normal school lives around the globe due to school closure and reclosure (United Nations, 2020a, 2020b). Studies have documented the negative consequences of school closure and lasting online learning in children, such as adverse physical, mental, and social well-being, poor educational outcomes, and broadened educational inequalities (Hoffman and Miller, 2020; Masonbrink and Hurley, 2020; UNICEF, 2020; Van Lancker and Parolin, 2020). Although studies that have abundantly examined the effect of COVID-19 on children’s education, a topic that has received comparatively much less attention is how the pandemic affects young children’s school readiness for the transition from kindergartens to elementary school.

School readiness refers to “the state of child competencies at the time of school entry that are important for later success” (Snow, 2006, p. 9). Nurturing children to be ready for school is vital, as cumulative evidence has shown that a good school readiness for formal education is linked to both short-term and long-term consequences, including better academic achievements, better mental health, less substance use, and less criminality (Trentacosta and Izard, 2007; McClelland et al., 2014; Jones et al., 2015; Lynch et al., 2017; Pan et al., 2019). Given the importance of school readiness in childhood, scholars have designed a number of intervention programs to enhance children’s school readiness (Sheridan et al., 2010; McClelland et al., 2019). However, promoting school readiness in children is not an easy task (Kagan and Neuman, 1997; Boivin and Bierman, 2014). This task has become even more challenging in the past year due to the outbreak of COVID-19, which has precluded a majority of offline activities that could have been originally arranged to promote school readiness (e.g., on-site visit). To our knowledge, little research has examined children’s school readiness and the role of parents in getting their children ready for school in time of COVID-19. A timely understanding of this topic is crucial to shed light on ways to promote children’s school readiness in this particular period.

## Hong Kong Children’s Learning During Covid-19 Outbreak

In Hong Kong, the formal education starts with primary school and kindergarten is not compulsory. Nevertheless, most children are enrolled in kindergarten because the educational system is competitive and parents have high expectations on their children’s early preparation for formal schooling (Lau, 2014; Wong and Rao, 2015; Lau and Ng, 2019). During 2020 and 2021, the Education Bureau of Hong Kong announced school suspension from time to time and advised to switch face-to-face learning activities to distance learning in response to the irregular outbreak of COVID-19. According to a large-scale survey (Lau and Lee, 2021), although most kindergartens and primary schools in Hong Kong responded rapidly by offering online courses, a number of concerning issues also emerged. First, the sudden change to distance learning increased teachers’ burden of making relevant materials. Worse still, most of the distance learning was not interactive and did not sufficiently meet parents’ expectations. Second, parents were required to help their kids with distance learning, especially for lower grade children. However, many parents did not have sufficient time and they lacked relevant knowledge to assist their kids’ learning. Third, a significant proportion of parents were dissatisfied with schools’ arrangements no matter whether distance learning was offered to their children, and the top reason of dissatisfaction was lack of support. These findings not only suggested that children’s learning and academic activities that are essential for school readiness had been largely disrupted, but also implied that parents’ perceived social support, their competence to help with their kids’ learning, and whether they had sufficient time with their children emerged as crucial factors associated with Hong Kong children’s learning as well as school readiness in times of COVID-19. Using survey data collected from Hong Kong parents, this research aims to understand Hong Kong children’s school readiness during the new normal period and the role of parent perceived social support, their competence, and time spent with children.

## Children’s School Readiness

Research on school readiness has generated a number of theoretical accounts in the last 50years. Early maturational perspective suggests that children’s development is preprogramed by their biological time clock and therefore children’s readiness to learn relies on their cognitive maturity level (cf. Kagan, 1992). In light of this view, deficits in school readiness lie within the child and the development of children cannot be pushed beyond their biological development level by teaching (Winter and Kelley, 2008). More recently, the holistic account suggests that both children’s skills (e.g., emotional and social skills) and the roles of ecological systems (e.g., family) should be emphasized (Diamond, 2010). Prior research found that teachers and parents typically defined school readiness in terms of children’s cognitive, self-care, psychomotor, language, and social–emotional skills and abilities (Altun, 2018). Besides, Blair, and colleagues (Blair, 2002; Blair and Raver, 2015) further considered that self-regulation should also be included as an additional component of school readiness, as it addresses children’s ability to attend to information, use it appropriately, and inhibit behavior that interferes with learning (Pan et al., 2019). Of note, the holistic perspective not only suggests that children’s school readiness can be measured in terms of various skills and abilities at the time of school entry (Snow, 2006), but it also informs that children’s school readiness can be improved both by strengthening children’s skills and abilities (e.g., McClelland et al., 2019) and by building an engaging environment that supports children’s learning and growth (e.g., Sheridan et al., 2010). Inspired by the holistic account, we measured children’s school readiness by asking parents to rate the extent to which their children were ready for primary school in terms of various skills and abilities (e.g., self-care, social, emotional, etc.).

## The Association Between Parent Perceived Social Support, Parent Competency, Time Spent with Children, and School Readiness

Parent perceived social support refers to parent’s overall perception of the tangible (e.g., money) and intangible (e.g., knowledge) resources for stress management within their social network (French et al., 2018). The social support parents receive is typically considered a distal predictor of child outcomes through parenting processes (Bronfenbrenner, 1979). Several studies have found that parent perceived social support is related to higher quality parenting practices in mothers (Shin et al., 2006; Wallace, 2013). Prior research also found a positive association between parent perceived social support and better cognitive and behavioral outcomes in children (Carothers et al., 2006). Using longitudinal data, Bono et al. (2016) found that parent perceived social support was both directly and indirectly (through depression and parenting behavior) related to children’s school readiness (measured as cognitive, language, and social–behavioral skills). In addition, parent perceived social support has been viewed as a protective factor that strengthens parents’ and children’s resilience in response to various stressors (Armstrong et al., 2005; Algood et al., 2013). This perspective aligns with the family stress model which suggests that protective factors may mitigate the negative impacts of parental stress and disrupted parenting on child adjustment outcomes (Masarik and Conger, 2017). However, a recent study employed latent profile analysis to examine the extent to which parent perceived social support was related to different school readiness profiles in black girls, but no significant findings were achieved (Iruka et al., 2020). Taken together, only a small number of studies have so far directly examined the association between parent perceived social support and children’s school readiness, and these limited studies were conducted in a non-pandemic period. Although a research discussed that parents perceived social support would be important to children’s home learning and mental health during the COVID-19 pandemic, it did not directly examine the association between parent perceived social support and children’s learning (Ren et al., 2020). In sum, little is known whether the existing findings could be generalized to this new normal period. Moreover, the mixed findings in the literature suggest that there might be certain factors moderating the said link, but scant research has examined this possibility. Hence, it is necessary to further reveal under which conditions social support parents perceive would have a larger effect on children’s school readiness. In this study, we examined whether parent competence and the quantity of time spent with the child might serve as potential moderators.

In this study, parent competence refers to the extent to which parent possess relevant knowledge, skills, and a positive attitude in helping with their children’s school transition. This definition well aligns with the building blocks of the Triple-P program (Sanders et al., 2003). With high competence, parents are more likely to have positive parent–child interactions (Moon et al., 2020). For instance, competent parents are able to provide clear expectations for child behavior, give positive reinforcement to consolidate desirable behavior, and engage their children in an interactive and developmentally appropriate interactions, all of which offering a facilitative environment for the development of school readiness skills, such as language, cognitive, and social–emotional skills (Burchinal et al., 2002). The role of parent competence in the association between parent perceived social support and children’s school readiness may be nuanced. On the one hand, according to the risk–resilience model (Masten, 2001), social support can be seen as the external resource that mitigates the effect of family risk (e.g., low parent competence) on child developmental outcomes (e.g., school readiness). In this sense, parent perceived social support could be positively related to children’s better school readiness when parents have low competence in scaffolding their children. On the other hand, parents with high competence are more likely to maximize the support services provided to them and better address their needs with the support (Scheel and Rieckmann, 1998; Harty et al., 2007; Lee et al., 2020). The nuanced role of parent competence may further depend on whether parents spend enough time with their children. In a recent study conducted among highly educated Korean parents (a proxy of parent competence), it found that main caregivers’ (usually mothers) positive parenting behavior was related to children’s school readiness only for those who had more time with their children (Suh, 2021). Although this study did not directly measure parent competence, it implies that when parents have sufficient time with their children, they might have more opportunities to apply their parenting practices as well as the social support in fostering children’s school readiness. Taken together, we consider that when parents have enough time with their children, social support may be useful in facilitating children’s school readiness for parents with both high and low competence, because they have more time to utilize the social support. Furthermore, the effectiveness of social support might be even larger for parents with high competence because they are more likely to know how to maximize the social support. For parents who do not have enough time with their children, they might have less time applying the social support and thus less likely help with children’s school readiness. In other words, both parent competence and time spent with the child may jointly moderate the association between parent perceived social support and children’s school readiness.

## The Current Study

As mentioned, only a limited number of studies have examined the association between parent perceived social support and children’s school readiness and little is known about the boundary variables that moderate the effectiveness of social support, especially in times of COVID-19. To address these gaps, this study examined two questions: (1) the extent to which social support would be related to children’s school readiness and (2) the extent to which parent competence and time spent with the child would jointly moderate the “parent perceived social support – children’s school readiness” link. Based on the literature reviewed above, we hypothesize that parent perceived social support would be related to better school readiness in children and that this link would be more pronounced for parents who have high competence and more time with children. In addition, we controlled for a range of demographic characteristics (e.g., child gender, family SES, parent marital status, parent age, etc.) to rule out their potential confounding effects on children’s school readiness.

## Materials and Methods

### Research Design

Data of this study were part of an online survey on parenting and school transition during COVID-19 in Hong Kong. After obtaining the approval from the Human Research Ethics Committee at the authors’ affiliated university, an online survey was conducted between May and June 2021, approximate 3months before children entered primary school. Two sampling strategies were employed in this study. First, a total population sampling strategy was utilized, in which all parents from Hong Kong kindergartens are eligible to participate. Specifically, school invitation email was sent to all kindergartens (around 1,000 kindergartens in total) in Hong Kong explaining the aim of the study. Kindergartens were invited to provide information of the contact person by completing a form in Qualtrics. A total of 58 kindergartens agreed to help disseminate the research invitation to families of their students. Parents who were interested to participate were invited to complete the consent form and survey through the link provided on the invitation letter. Second, the study recruited a convenience sample *via* a Facebook page with parents as the target audience. The parent invitation letter, consent form, and online survey were provided to all participants through a hyperlink in the Facebook post. All participating parents completed the online parent survey *via* Qualtrics. Data obtained were exported from Qualtrics to SPSS for data analysis.

### Participants

A total of 825 parents consented to participate in this study. However, 106 parents who failed to complete the whole survey and 76 parents who reported their children as diagnosed with a special need were excluded from the analyses. Therefore, participants of the final sample were 643 parents recruited through 58 kindergartens (69.7%) and the Facebook (30.3%). These parents had children (*M*_age_=72.11months, SD=5.16months; 46.3% boys) who were in the final year of kindergarten (Table 1). Most of the respondents were mothers (87.1%), married (91.8%), and reported an age range of 36–40years old (33.3%). Most of the participating parents had a child transitioning to primary school for the first time (68.7%) and attending half-day kindergarten program (85.2%) at the time of the survey. The median range of monthly household income was HK$20,001–40,000 (US$1=HK$7.78; the median income of all Hong Kong families: HK$26,600, Census and Statistics Department, 2021).

**Table 1:** Demographic Variables of Study Population.

Variables	*n*	%
**Gender of child**		
Male	298	46.3
Female	345	53.7
**Gender of parent**		
Male	83	12.9
Female	560	87.1
**Marital status**		
Married	590	91.8
Other	53	8.2
**Age of parent**		
≤30	52	8.1
31–35	200	31.1
36–40	214	33.3
41–45	134	20.8
≥46	43	6.7
**First primary school transition of the child**		
Yes	442	68.7
No	201	31.3
**Type of class mode attended**		
Full day	95	14.8
Half day	548	85.2
**Monthly household income (in Hong Kong dollar)**		
<$10,001	42	6.5
$10,001–$20,000	183	28.5
$20,001–$40,000	206	32.0
$40,001–$60,000	99	15.4
$60,001–$80,000	52	8.1
$80,001–$100,000	28	4.4
>$100,000	33	5.1

### Instrumentation

The online survey was co-created by the authors based on their research expertise and research questions, as well as a careful review of relevant literature. The items related to “perceived support” and “time spent” with children were designed to directly address the constructs using a single item. On the other hand, multiple items were developed to assess the multidimensional nature of “parent competence” and “school readiness.” A pilot study was conducted with a total of 10 parents. The feedback received were related to the enhancement of the clarity and structure of the survey. Based on the feedback, relevant changes were made before the survey was finalized and used in the actual study. The online survey included questions in three main sessions: (1) parent–child relationships, (2) parents’ involvement and support for school transition, and (3) children’s school readiness outcomes. In this study, the following items were selected to address the research questions.

Perceived support (1 item): “Do you have enough support to help you with your child’s transition to primary school?” This item was rated on a four-point scale (1=*completely not enough*, 2=*a little bit enough*, 3=*moderately enough*, 4=*completely enough*).

Parent competence (three items): “Are you confident in helping your child’s primary school adaptation?” “Do you have the relevant skills to help your child adapt to primary school life?” and “Do you have the relevant knowledge to help your child adapt to primary school life?” on a four-point scale (i.e., 1=Completely not confident/knowledgeable/enough, Completely not confident/knowledgeable/enough 2=*a little bit confident/knowledgeable/enough*, 3=*moderately confident/knowledgeable/enough*, and 4=*very confident/knowledgeable/enough*). The three items assessed the parents’ efficacy, knowledge, and skill related to their competence in helping children’s primary school adaptation. These items were developed based on the parenting behaviors/practices scientifically demonstrated to be crucial for child developmental outcomes (Sanders et al., 2003; Dix and Meunier, 2009). The three items were averaged and the mean score was used as an indicator of parent competence. The internal consistency for the three items was 0.79.

Time spent with the child (one item): “Compared to the time before the pandemic, is there a change in the quantity of time spent with your child?.” This item was rated on a five-point scale (1=*a lot less*, 2=*a little bit less*, 3=*not much change*, 4=*a little bit more*, 5=*a lot more*).

School readiness (10 items): “How prepared is your child in the following skills for transition to primary school? (1) Self-care ability, (2) Social skills, (3) Emotion skills, (4) Motor ability, (5) Language ability, (6) Cognitive ability, (7) Mental preparation, (8) Academic knowledge, (9) Self-management, (10) Routine behavior.” These school readiness items were developed based on the skills considered important for school in both international and local contexts (e.g., Lau et al., 2011; Ip et al., 2016; Lau and Power, 2018). These items were rated on a four-point scale (1=*completely not prepared*, 2=*not prepared*, 3=*prepared*, and 4=*completely prepared*). The internal consistency for the ten items was 0.89.

### Data Analysis

We analyzed the data in SPSS 26.0 (IBM Corp, 2019) in several steps, with 0.05 as the level of significance. First, we examined the mean levels of each school readiness indicator to understand the extent to which Hong Kong parents perceived their child was ready for transition to primary school. Repeated measure ANOVA was carried out to examine whether parents’ ratings on each school readiness indicator were significantly different among each other. Pairwise comparisons were followed and corrected level of significance (i.e., 0.05/10=0.005) was applied to determine the significant differences between each pair of indicators to control for Type I error. Second, we conducted descriptive statistics (e.g., means, standard deviations, and skewness) to capture the centralities of the main variables. Third, we performed bivariate correlations between parent perceived support, parent competence, time spent with the child, and children’s school readiness. The correlation coefficients were also interpreted in terms of effect sizes, with 0.10, 0.30, and 0.50 representing small, medium, and large effect sizes (Cohen, 1992). Fourth, we utilized Hayes’ (2017) PROCESS macro (version 3.15, Model, 3) to conduct a regression-based moderation model to examine the association between parent perceived support and children’s school readiness as well as the joint moderation effects of parent competence and time spent with the child. In this model, we tested three main effects (i.e., parent perceived support, parent competence, and time spent with child), three two-way interaction effects (i.e., parent perceived support * parent competence; parent perceived support * time spent with child, and parent competence * time spent with child), and one three-way interaction effect (i.e., parent perceived support * parent competence * time spent with child). Of note, the independent variable and the two moderators were centered and bootstrapping technique (*N*=5,000) was used. In the final step, we performed simple slope tests to examine the associations between parent perceived support and children’s school readiness by various combinations of the two moderators (i.e., low parent competence + less time with the child, low parent competence + more time with the child, high parent competence + less time with the child, and high parent competence + more time with the child).

## Results

### Levels of Children’s School Readiness

**Figure 1:**
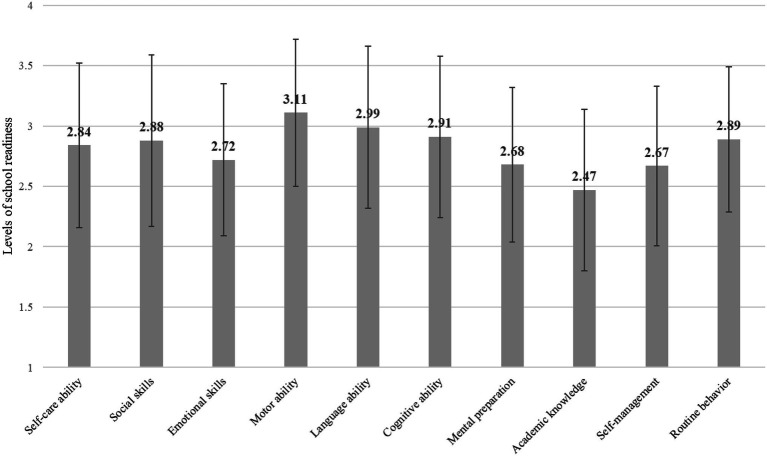
Levels of Different School Readiness Indicators.

As shown in Figure 1, Hong Kong parents had different ratings on various school readiness indicators. Specifically, parents considered that their children were quite ready in terms of motor ability (3.11 out of 4), language ability (2.99 out of 4), cognitive ability (2.91 out of 4), routine behavior (2.89 out of 4), and social skills (2.88 out of 4), whereas they perceived that their children were less prepared for primary school in terms of academic knowledge (2.47 out of 4), self-management (2.67 out of 4), and mental preparation (2.68 out of 4).

We conducted a repeated measures ANOVA to examine whether parents’ ratings on each school readiness indicators were different. The results of multivariate tests were significant, Wilk’s *Lambda*=0.476, *F*(9, 729)=73.61, *p*<0.001, *partial eta squared*=0.48, suggesting that significant differences were found for parent ratings on different school readiness indicators. The results of further pairwise comparisons are summarized in Table 2. In general, parent postulated that their children were most ready in terms of motor ability and that their children were least ready in terms of academic knowledge.

**Table 2:** Pairwise Comparisons of School Readiness Indicators.

School readiness indicators	Pairwise Comparison (I-J Difference)
1. Self-care ability	1–2 (−0.02); **1–3 (0.11)**, **1–4 (−0.29)**, **1–5 (−0.13)**, 1–6 (−0.07), **1–7 (0.15)**, **1–8 (0.34)**, **1–9 (0.17)**, 1–10 (−0.05)
2. Social skills	**2–3 (0.13)**, **2–4 (−0.27)**, 2–5 (−0.10), 2–6 (−0.05), **2–7 (0.17)**, **2–8 (0.36)**, **2–9 (0.19)**, 2–10 (−0.02)
3. Emotional skills	**3–4 (−0.40)**, **3–5 (−0.23)**, **3–6 (−0.18)**, 3–7 (0.04), **3–8 (0.23)**, 3–9 (0.06), **3–10 (−0.15)**
4. Motor ability	**4–5 (0.16)**, **4–6 (0.22)**, **4–7 (0.44)**, **4–8 (0.63)**, **4–9 (0.45)**, **4–10 (0.24)**
5. Language ability	5–6 (0.05), **5–7 (0.27)**, **5–8 (0.47)**, **5–9 (0.29)**, 5–10 (0.08)
6. Cognitive ability	**6–7 (0.22)**, **6–8 (0.41)**, **6–9 (0.24)**, 6–10 (0.03)
7. Mental preparation	7**–8 (0.19)**, 7–9 (0.02), **7–10 (−0.19)**
8. Academic knowledge	**8–9 (−0.18)**, **8–10 (−0.39)**
9. Self-management	**9–10 (−0.21, *p* <0.001)**
10. Routine behavior	-

*Pairwise comparisons that are significant at the corrected level of significance (*i.e.*, p<0.005) are bolded. Positive and negative values of the I-J difference indicates that the value of I is significantly larger and smaller than the value of J, respectively*.

### Means, Standard Deviation, and Bivariate Correlations Among the Main Study Variables

In general, parents perceived inadequate support for their child’s transition to primary school (2.02 out of 4), and they also felt that they were not sufficiently competent of assisting their child (2.11 out of 4). In the meanwhile, they spent as much time with their child in times of COVID-19 as the days before the outbreak. In addition, they perceived that their child was moderately ready for primary school overall (2.81 out of 4). Regarding the bivariate correlations, the results showed that high levels of perceived support, parent competence, and time spent with the child were positively related to children’s school readiness at about medium, medium-to-large, and small effect sizes, respectively (Table 3).

**Table 3:** Means, Standard Deviations (SD), and Bivariate Correlations among the Main Study Variables.

Variables	1	2	3	4
1. Parent perceived support	-			
2. Parent competence	0.63^***^	-		
3. Time spent with the child	0.02	0.11^**^	-	
4. Child school readiness	0.37^***^	0.46^***^	0.13^**^	-
Range	1–4	1–4	1–5	1–4
M	2.02	2.11	3.61	2.81
SD	0.75	0.62	0.90	0.47
Skewness	0.58	0.57	0.04	−0.22

** *p<0.01*;

*** *p<0.001*.

### The Moderation Analysis of the Association Between Parent Perceived Support and Children’s School Readiness by Parent Competence and Time Spent With the Child

The overall model accounted for 27.0% variance of children’s school readiness, and the results are summarized in Table 4. As shown, child gender and whether the target child was the first one at home for school transition were significantly related to children’s school readiness, with girls and the target child being the first one for school transition reported to be more ready.

**Table 4:** Moderation Analysis of the Association between Parent Perceived Support and Child School Readiness by Parent Competence and Time Spent with the Child.

Predictors	*B*	*SE*	*p*	*95% CI*
Report informant (1=mother, 2=father)	0.07	0.05	0.187	[−0.03, 0.17]
Child gender (1=boy, 2=girl)	**0.08**	**0.03**	**0.009**	**[0.02, 0.15]**
Class mode (1=full day, 2=half day)	−0.03	0.05	0.491	[−0.12, 0.06]
First child in transition (1=yes, 2=no)	**−0.10**	**0.04**	**0.005**	**[−0.18, −0.03]**
Marital status (1=married, 2=other)	0.07	0.06	0.228	[−0.05, 0.19]
Parent age	−0.01	0.02	0.598	[−0.05, 0.03]
Family monthly income	−0.01	0.01	0.351	[−0.03, 0.01]
Parent perceived support	**0.10**	**0.03**	**0.001**	**[0.04, 0.15]**
Parent competence	**0.31**	**0.04**	**< 0.001**	**[0.24, 0.38]**
Time spent with the child	0.03	0.02	0.191	[−0.01, 0.07]
Parent perceived support * parent competence	−0.05	0.03	0.102	[−0.10, 0.01]
Parent perceived support * time spent with the child	0.03	0.03	0.282	[−0.03, 0.10]
Parent competence * time spent with child	**−0.11**	**0.04**	**0.003**	**[−0.18, −0.04]**
Parent perceived support * parent competence * time spent with the child	**0.06**	**0.03**	**0.038**	**[0.003, 0.12]**

*Bold values are the significant effects*.

After controlling for demographic covariates, the main effect of parent perceived support (*B*=0.10, *p*=0.001) and parent competence (*B*=0.31, *p*<0.001) was significant, but the main effect of time spent with the child was not (*B*=0.03, *p*=0.191). The two-way interaction effect between parent perceived support and parent competence (*B*=−0.05, *p*=0.102) and the one between parent perceived support and time spent with the child (*B*=0.03, *p*=0.282) was not significant, but the two-way interaction effect between parent competence and time spent with child was (*B*=−0.11, *p*=0.003). More importantly, the three-way interaction was also found significant (*B*=0.06, *p*=0.038).

Breaking down the three-way interaction effect, we found that the interaction between parent perceived support and parent competence was only significant when parents spent less time (−1 SD) with their children (*B*=−0.10, *p*=0.017) but not when they spent more time (+1 SD) with their children (*B*=0.08, *p*=0.820).

**Figure 2:**
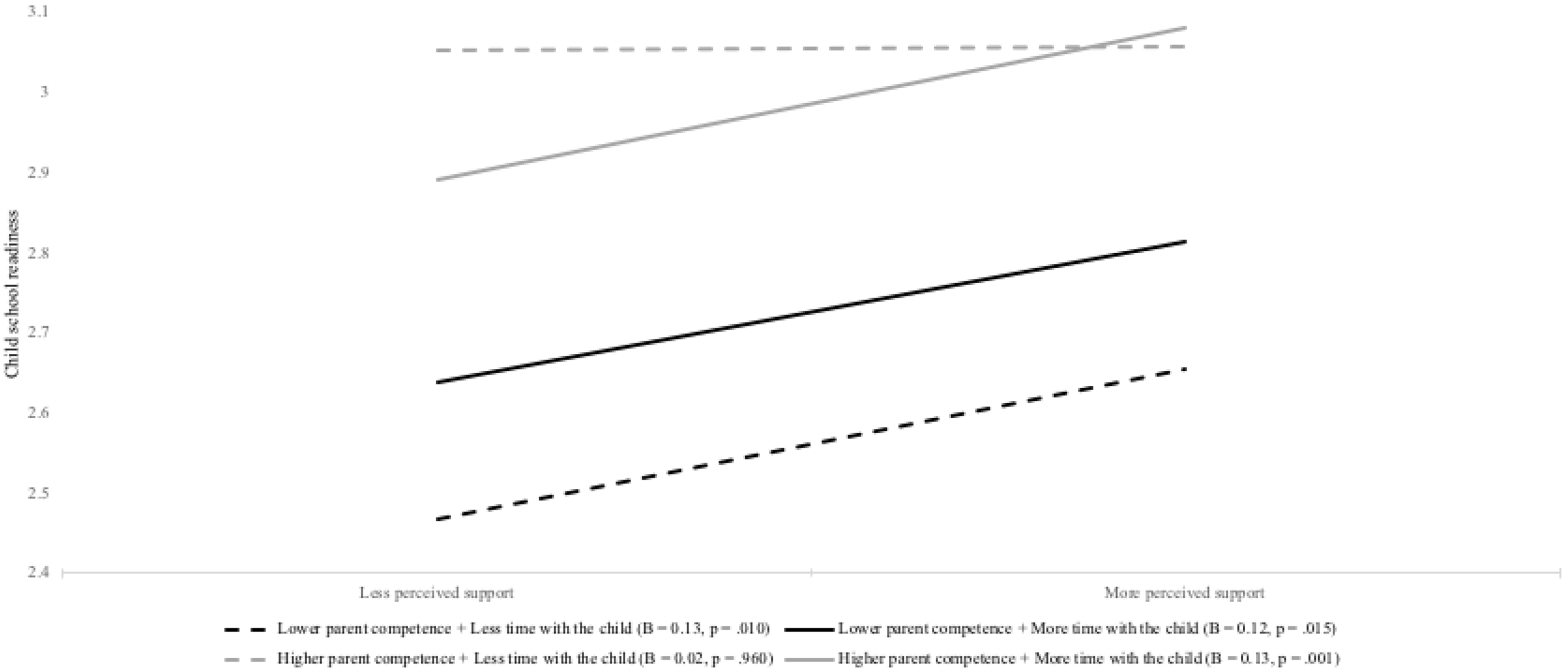
Simple Slope Tests for the Association between Parent Perceived Support and Child School Readiness by Parent Competence and Time Spent with the Child. Lower/less and high/more level refers to 1 SD below and above the mean of the variable, respectively.

We further broke down the two-way interaction effect between parent perceived support and parent competence and examined the simple slopes for the association between parent perceived support and children’s school readiness by the four combinations of the two moderators. The results are summarized in Figure 2. For parents who reported lower competence and spent less time with the child, higher levels of perceived support were related to stronger children’s school readiness (*B*=0.13, *p*=0.010, 95% CI=[0.03, 0.23]). For parents who reported lower competence and spent more time with the child, higher levels of perceived support were also related to child stronger school readiness (*B*=0.12, *p*=0.015, 95% CI=[0.02, 0.22]). For parents who reported higher competence and spent less time with the child, higher levels of perceived support were *not* related to children’s school readiness (*B*=0.02, *p*=0.960, 95% CI=[−0.09, 0.10]). For parents who reported higher competence and spent more time with the child, higher levels of perceived support were related to stronger children’s school readiness (*B*=0.13, *p*=0.001, 95% CI=[0.05, 0.21]). Taken together, these findings suggested that children’s school readiness was the joint function of perceived support, parent competence and how much time parents spent with the child.

## Discussion

The existing studies have widely examined the effect of COVID-19 on children’s developmental outcomes, such as well-being, educational achievements, and educational inequalities (Hoffman and Miller, 2020; Masonbrink and Hurley, 2020; UNICEF, 2020; Van Lancker and Parolin, 2020), but little is known how well preschoolers are ready for school in time of COVID-19 and the role of parents in promoting children’s school readiness. Given the importance of children’s school readiness to their short-term and long-term developmental outcomes, nurturing children to be ready for formal schooling is highly necessary and significant. During COVID-19, many offline activities that could have been carried out to foster children’s school readiness have to be canceled. At the time of writing, several organizations have provided various forms of support to facilitate Hong Kong children to transition to primary school (e.g., The Education University of Hong Kong, 2021). However, little research has examined the extent to which parent perceived social support is linked to children’s school readiness and what factors moderate the effectiveness of the social support. In this study, we examined the association between parent perceived social support and children’s school readiness and the moderation of parent competence and time spent with the child. The current findings largely confirm our hypotheses, suggesting that Hong Kong children’s school readiness in times of COVID-19 is a function of parent perceived social support, parent competence, and the quantity of parent–child time.

Descriptive findings indicated that Hong Kong parents did not receive adequate social support. This result is consistent with Lau and Lee’s (2021) research findings that Hong Kong parents were not satisfied with school’s distance learning arrangement and would like to have more support for their children’s learning during the outbreak of COVID-19 in 2020. In particular, parents rated their children to be least ready in terms of academic knowledge. This finding may reflect Hong Kong parents’ high expectations on their children’s preparedness for formal schooling in order to fit in the school soon and achieve good academic accomplishment (Wong and Rao, 2015; Lau and Ng, 2019).

Consistent with prior research (Bono et al., 2016) and discussion (Ren et al., 2020), our findings indicate that parent perceived social support is positively related to better school readiness in children even in times of COVID-19. The current results support the view that the parent perceived social support parents is a crucial resource for them to deal with the stressors (Armstrong et al., 2005; Algood et al., 2013), such as promoting children’s learning activities even in times of COVID-19 (Oppermann et al., 2021).

Going beyond and above, the significant findings of moderation analyses suggest very nuanced association between parent perceived social support and children’s school readiness. For parents who have more time with their children, the “parent perceived social support – school readiness” link is significant both for parents with higher and lower competence, but perhaps due to *different underlying mechanisms*. For parents with higher competence, the reason why perceived social support is useful in facilitating children’s school readiness might be because these parents know better how to maximize the utility of the social support (Scheel and Rieckmann, 1998; Harty et al., 2007; Lee et al., 2020). For parents with lower competence, perceived social support is useful because it may compensate the family inadequacies, which is consistent with the risk-resilience model (Masten, 2001) and prior research which discloses that using intervention to enhance the family environment can benefit children’s school readiness (Sheridan et al., 2010). Of note, although these explanations appear plausible, we did not carry out direct examination and it would be promising for future research to investigate these potential mechanisms more closely.

Under the condition that parents have less time with their children, parent perceived social support is only effective for parents with lower rather than higher competence. This result is somewhat consistent with Suh’s (2021) study which found that competent mothers’ positive parenting behavior was positively related to their children’s school readiness for those who could spend more time with children. One plausible explanation for these findings is that when parents do not feel competent, they might be more likely to just follow the instructions of the social support. By contrast, for competent parents, they might not be content of just utilizing the social support but might also be prone to figure out the rationale of the social support and how to use the social support better. Without enough time to go through the deliberative processes, competent parents might possibly feel social support is not empowering enough and might be reluctant to fully utilize the support, thus rendering the perceived social support much less effective. This explanation somewhat concurs with a recent study which found that parents who felt competent to help with their kids’ difficulties were less likely to simply utilize social support because it might be a sign of inability to competent parents (Benatov, 2019). Given the scarce volume of this line of research, it would be promising for future studies to examine how parents with high and low competence select and utilize social support differently to promote their children’s school readiness.

## Implications

This study offers important insight into children’s school readiness in times of COVID-19. Specifically, the findings that parents did not receive adequate support and that parents’ perceived social support is associated with better children’s school readiness call for more support for parents during the pandemic to foster their children’s school readiness. Importantly, the findings that perceived social support is associated with children’s school readiness among the groups of parents who can spend more time with their children, regardless of their perceived competence, and parents who had lower competence and spent less time with children suggested that universal support should be provided to parents regardless of whether they are able to spend more time with their children and their perceived competence.

Specifically, traditional school transition support practices for families, including providing information about primary schools and expectations for children’s behavior, suggested activities for parent–child interaction that promotes children’s school readiness skills, and advice on parenting strategies that would promote children’s adjustment during school transition should continue to be implemented during the pandemic. Although school closures and the use of distance learning may be necessary during the pandemic in different countries, appropriate and adequate support must be provided to parents to prepare their children for the transition to primary schools as far as practical in a safe manner during the pandemic (e.g., conducting parent education online, implementing school transition support programs in small groups, and providing detailed guidelines for preparing children).

On the other hand, the finding that social support was not associated with children’s readiness for school among parents who spent less time with their children but have high competence suggested that tailored support should be provided to this group of parents. This group of parents are likely to be busy parents with high parental efficacy. As a result, they may not have time to involve or may not see the need of learning new parenting skills from their social support, even if social support is adequately available. To address their needs, developing tailored, easy-to-follow steps and involvement strategies that are evidence-based may be useful to encourage parental involvement and the use of effective strategies to help children get ready for primary school. As such, efforts are needed to improve schools’ and community’s ability to support parents and students, in which professional development training should be provided to teachers and different family service providers to increase their capacity in supporting parents and respond rapidly to challenges related to school transition during the pandemic in a sensitive way.

## Limitations and Future Directions

The present study has the following limitations. Most notably, this study only collected data at one time point, and thus, longitudinal and causal relations between parent perceived social support and child school readiness cannot be ascertained. Future studies should collect data across multiple time points to confirm the influence of social support on children’s readiness outcomes over time. Second, the data were only collected in Hong Kong where the early education systems are quite unique with high parental expectation for academic performance and high parental involvement in children’s learning. Thus, the findings and implications may be culturally specific and cannot be generalized to other cultural contexts. Future studies should examine the issue using a non-Hong Kong sample in different cultural contexts to inform the design of support for parents for promoting children’s school readiness during the pandemic. Finally, the present study utilized parent reports for assessment of all main variables and the findings may be biased as a result of the informant bias. Future research should consider utilizing multi-informants (e.g., child and spouse reports, observation), more standardized measures, and objective methods (e.g., child tests) in their assessments.

## Conclusion

To our knowledge, this study is among the first to explore the association between parent perceived social support and children’s school readiness, as well as the moderating effects of parent competence and time spent with children in such relation. Findings suggested that the support parents receive for preparing their child for primary school is inadequate during the COVID-19 pandemic and that children are not completely ready for school. Findings from moderation analyses also suggested that parent perceived social support is positively related to children’s school readiness, except for the group of children whose parents have less time with them and have higher competence. Together, the results call for more general support for parents so that they can better prepare their children for primary school during the pandemic. The results also suggested the importance of providing tailored support to parents who have less time with their children and have higher competence to promote their child’s school readiness.

## Data Availability Statement

The raw data supporting the conclusions of this article will be made available by the authors, without undue reservation.

## Ethics Statement

The studies involving human participants were reviewed and approved by The Education University of Hong Kong. The patients/participants provided their written informed consent to participate in this study.

## Author Contributions

EL and J-BL contributed to conception and design of the study. EL organized the database. J-BL performed the statistical analysis. EL and J-BL wrote the first draft of the manuscript. All authors contributed to manuscript revision, read, and approved the submitted version.

## Conflict of Interest

The authors declare that the research was conducted in the absence of any commercial or financial relationships that could be construed as a potential conflict of interest.

## Publisher’s Note

All claims expressed in this article are solely those of the authors and do not necessarily represent those of their affiliated organizations, or those of the publisher, the editors and the reviewers. Any product that may be evaluated in this article, or claim that may be made by its manufacturer, is not guaranteed or endorsed by the publisher.
